# Clinical characteristics and outcomes in HIV-associated diffuse large B-cell lymphoma in China: A retrospective single-center study

**DOI:** 10.7150/jca.51027

**Published:** 2021-03-15

**Authors:** Jiazhu Wu, Yi Miao, Chuan Qian, Pengfei Tao, Xicheng Wang, Xingqi Dong, Xia Li, Jincheng Lou, Jinhua Liang, Wei Xu, Jianyong Li, Haiyan Min

**Affiliations:** 1 Department of Hematology, the First Affiliated Hospital of Nanjing Medical University, Jiangsu Province Hospital, Nanjing, 210029, China.; 2 Key Laboratory of Hematology of Nanjing Medical University, Nanjing, 210029, China.; 3 Department of Infectious Diseases, Yunnan Provincial Infectious Diseases Hospital/Yunnan AIDS Care Center, Kunming, 650000, China.

**Keywords:** diffuse large B-cell lymphoma, HIV, outcomes

## Abstract

Human immunodeficiency virus (HIV) infection is associated with an increased risk of aggressive lymphoma, especially diffuse large B cell lymphoma (DLBCL). There are few data regarding HIV-associated DLBCL in China. Therefore, we analyzed the characteristics and outcomes of patients with HIV-associated DLBCL from our center. We retrospectively studied HIV-infected patients with DLBCL from 2011 to 2019. Data on HIV infection and lymphoma characteristics, treatments and outcomes were retrieved and analyzed. In 78 patients with HIV-associated DLBCL, most had poor performance status (PS) (74%), elevated lactate dehydrogenase (LDH) levels (95%), B symptoms (74%), advanced Ann Arbor stages (81%), bulky diseases (64%) and extranodal involvement (70%) at diagnosis. The median CD4^+^ T cell count was 162/µl, and 26 patients were already on combination antiretroviral therapy (cART) treatment at diagnosis of DLBCL. Elevated whole blood EBV DNA copy number was detected in 38 patients (66%, 38/58). Of the 45 patients evaluated at the end of treatment, 26 (58%) achieved CR, 6 (13%) achieved PR and 6 (13%) experienced progressive disease. The 2-year progression-free survival (PFS) and overall survival (OS) rates were 56.4% and 62.7%, respectively. Factors associated with decreased PFS and OS in univariate analysis were unfavorable PS and high international prognostic index. Elevated EBV DNA copy number was inclined to be associated with worse outcome. We did not observe a significant difference in survival between R-EPOCH and R-CHOP regimens. In our population, patients with HIV-associated DLBCL presented with aggressive characteristics and exhibited poor survival outcomes, even in the modern cART era.

## Introduction

Human immunodeficiency virus (HIV) infection is associated with an increased incidence of malignancy. HIV-associated diffuse large B cell lymphoma (DLBCL) and Burkitt's lymphoma (BL) are among the most common cancers in HIV-positive patients 1-3. Other non-Hodgkin's lymphomas (NHLs), including primary effusion lymphoma (PEL), plasmablastic lymphoma, KSHV-associated multicentric Castleman's disease, primary central nervous system (CNS) lymphoma and classic Hodgkin's lymphoma (cHL), tend to be diagnosed in HIV patients as well 1. HIV strongly contributes to NHL mortality, particularly in acquired immunodeficiency syndrome (AIDS)-defining subtypes 4. With the introduction of combination antiretroviral therapy (cART), the incidence of HIV-associated lymphoma has decreased, and survival outcome have improved 4, 5.

DLBCL is the most common subtype of lymphoid malignancy in adults. It represents approximately 30% of NHL cases without HIV infection and 45% of cases with HIV-related lymphoma 6-8. In a Spanish study, compared to HIV-uninfected DLBCL patients, HIV-infected patients presented more aggressive features with a poorer performance status, more frequent B symptoms and more advanced Ann Arbor stages 5. In the above study, when treated with the standard-of-care regimen rituximab plus cyclophosphamide, hydroxydaunorubicin, vincristine and prednisolone (R-CHOP), HIV-infected patients exhibited similar disease-free survival but significantly worse overall survival (OS) compared to those without HIV infection 5. In contrast, a French study revealed that HIV-infected patients with DLBCL showed survival outcomes similar to those of HIV-negative DLBCL patients 9.

Infections with Epstein-Barr virus (EBV), hepatitis B virus (HBV), and/or hepatitis C virus (HCV) have been previously reported in DLBCL, and the former two viruses independently predict poor prognosis 10-13. In HIV-infected patients with DLBCL, it has been shown that elevated EBV load predicts inferior survival outcome; however, few data on HIV and HBV/HCV coinfection in patients with DLBCL have been published 13.

The HIV epidemic appears to have an obvious regional distribution in China, occurring frequently in Sichuan Province and Yunnan Province 14, 15. To date, only a few studies have described the clinical features of Chinese AIDS patients with DLBCL 16, 17. Herein, we retrospectively analyzed the characteristics and outcomes of Chinese HIV-infected DLBCL patients in the Yunnan Provincial Infectious Diseases Hospital/Yunnan AIDS Care Center.

## Methods

### Patients

A total of 104 cases of HIV-infected individuals with DLBCL were newly diagnosed between 2011 and 2019 at the Yunnan Provincial Infectious Diseases Hospital/Yunnan AIDS Care Center, which is the largest HIV/AIDS referral hospital in Southwest China. This study was approved by the institutional review board of Yunnan Provincial Infectious Diseases Hospital and was conducted according to the Declaration of Helsinki. DLBCL diagnosis was established in accordance with the 2008 WHO classification and was classified using the Hans classification algorithm. Clinical data were collected from the medical records. HIV-infected patients diagnosed with DLBCL who did not receive chemotherapy were excluded (n=25). After one patient was excluded due to treatment with GemOx (gemcitabine and oxaliplatin) regimen, 78 patients treated with CHOP like regimen with or without rituximab (CHOP ± R) or EPOCH (etoposide, prednisone, vincristine, cyclophosphamide and doxorubicin) regimen with or without rituximab (EPOCH ± R) were finally included in the analysis. Data on patient demographic characteristics (gender and age), HIV-related characteristics (HIV transmission route, years of HIV infection at DLBCL diagnosis, CD4^+^ cell count at DLBCL diagnosis and date of cART initiation), lymphoma-related characteristics (cell-of-origin subtype, Eastern Cooperative Oncology Group (ECOG) performance status score, serum lactate dehydrogenase (LDH), B symptoms, extranodal sites, Ann Arbor stage, international prognostic index (IPI) score, bulky tumor, Ki 67, BCL-2 expression by immunohistochemistry, bone marrow involvement and CNS-IPI score) and other related characteristics (EBV load, HBV load, HCV load, comorbidities, time from first complain to diagnosis) were available.

In this study, the cART regimen included two nucleoside reverse transcriptase inhibitors and one nonnucleoside reverse transcriptase inhibitor. After the diagnosis of DLBCL was initiated, patients were switched to cART treatment with tenofovir, lamivudine, and efavirenz or raltegravir potassium. Patients with a CD4^+^ cell count below 200/μl received trimethoprim/sulphamethoxazole as a prophylactic agent against *Pneumocystis jirovecii*. HIV-1 RNA load was monitored during chemotherapy. For patients with positive hepatitis B surface Ag (HBsAg), prophylaxis was not used because both tenofovir and lamivudine have antiviral activity against HBV. HBV DNA (cutoff ≥ 5×10^2^ copies/ml) and HCV RNA loads (cutoff ≥ 5×10^2^ IU/ml) were assessed during chemotherapy as well. Laboratory monitoring of plasma EBV DNA (cutoff ≥ 5×10^3^ copies/ml) load was performed in some patients.

### Response assessment

Interim evaluation was performed after completion of three or four cycles of chemotherapy. One month after completion of all treatments, the efficacy was evaluated. Computed tomography (CT) or ^18^F-fluorodexyglucose positron emission tomography (PET) was performed for radiological evaluation. Brain magnetic resonance imaging (MRI) was used to assess CNS involvement. The 2007 revised Cheson criteria were performed to define complete response (CR), partial response (PR), progressive disease (PD) and relapse.

### Statistical analysis

All statistical data were analyzed using SPSS software, version 21 or GraphPad Prism 8. Continuous variables are presented as the median with the first and third quartiles, and categorical variables are presented as numbers and percentages. Progression-free survival (PFS) was defined as the time from DLBCL diagnosis to progression, relapse or death from any cause. Overall survival (OS) was defined as the time from DLBCL diagnosis to last follow-up or death from any cause. Survival curves were plotted using the Kaplan-Meier method, and the log-rank test was used for comparison. Cox proportional regression models were performed for univariate and multivariate analyses of outcomes. A P-value <0.05 was considered statistically significant.

## Results

### Patient characteristics

A total of 78 patients were included in this analysis. Baseline clinical features of HIV-infected patients are summarized in Table 1. Of these patients, the median age was 43, and 67 (86%) were male. The main HIV transmission group was heterosexuals. Among 78 patients, 29 had a known HIV infection history of more than 1 year at the time of DLBCL diagnosis, and in 37 patients, DLBCL and HIV infection were diagnosed concomitantly (difference less than three months). The median CD4^+^ T cell count at DLBCL diagnosis was 162/μl (range 6-559) in these 78 patients, of whom 10 had a CD4^+^ T cell count less than 50/μl. At DLBCL diagnosis, 26 patients were already on cART treatment. The median time from patients' first complaint to diagnosis was 2 months. Most patients (74%) had a poor performance status (PS) (ECOG PS 2-4), and elevated LDH at diagnosis was present in 95% of patients. Seventy-four percent of patients had B symptoms, 81% had an advanced Ann Arbor stage (III-IV), and 64% had bulky tumors at diagnosis. Fifty-five patients (70%) exhibited extranodal involvement, of whom twenty-six possessed more than 2 extranodal sites. The most frequent extranodal sites were the gut (n=30), stomach (n=12), liver (n=10), bone (n=7), pancreas (n=7) and/or kidney (n=6). One-third of patients exhibited a higher central nervous system IPI (CNS-IPI) at diagnosis. A Ki-67 proliferation index higher than 90% was observed in 22 cases. Overall, 30 samples (39%) showed positive BCL2 expression in tumor cells (>50%), 29 (37%) were negative and 19 (24%) were unknown. Among 58 cases with information on EBV status, EBV load was elevated (5×10^3^ copies/ml) in 38 (66%). Of all patients, eight (10%) presented with positive HBsAg and eight (10%) with positive anti-HCV antibody.

### DLBCL treatment

Fifty-three (53/78) patients received the CHOP±R regimen, among whom twenty-five received six to eight cycles. Twenty-four of these 53 patients received R-CHOP, twenty-one received the CHOP regimen, and five received CHOP followed by R-CHOP or vice versa, depending on their financial situation at that time. Two patients received first-line R-CHOP, progressed at interval evaluation; one was changed to the GemOx regimen, and the other was switched to the R-GDP (gemcitabine, dexamethasone and carboplatin) regimen. One patient who received 4 cycles of R-CMOP (cyclophosphamide, mitoxantrone, vincristine and prednisone) was switched to R-GOD (gemcitabine, oxaliplatin and dexamethasone) plus oral lenalidomide due to PD. Twenty-five patients (age ≤60) with high risk (aaIPI scores 2-3) received regimens, including the DA-EPOCH±R (etoposide, prednisone, vincristine, cyclophosphamide and doxorubicin) regimen. Among these, 9 patients received the R-DA-EPOCH regimen, and 13 patients received one or two cycles of R-CHOP followed by R-DA-EPOCH. Fifty-six of 78 patients received intrathecal methotrexate and cytarabine for central nervous system prophylaxis. Radiotherapy with 40-50 Gy was given in eleven patients with bulky tumors, among whom 4 patients received radiotherapy as consolidation after chemotherapy with CR, and 7 were treated after four cycles of chemoradiotherapy with additional cycles afterwards. Five patients were given autologous hematopoietic stem cell transplant (autoSCT) following chemotherapy.

### Treatment response and outcomes

Among sixty-seven HIV-infected DLBCL patients who were evaluable in the interim analysis, the overall response rate (ORR) was 88%, including 14 (21%) CR and 45 (67%) PR. Three patients experienced PD, and five patients died before the interval evaluation. One died from hemorrhage, two from serious infection and two from rapid disease progression. Of the 45 patients evaluated at the end of treatment, 26 (58%) achieved CR, 6 (13%) achieved PR and 6 (13%) experienced progressive disease (Table 2). The other 7 patients died during chemotherapy before response could be evaluated from sepsis (n=4), rapid disease progression (n=2) or heart failure (n=1). No one included in our cohort presented with baseline CNS involvement. With over 70% of patients receiving CNS prophylaxis, only one patient experienced CNS relapse 4 months after reaching CR and died soon thereafter.

**Figure 1:**
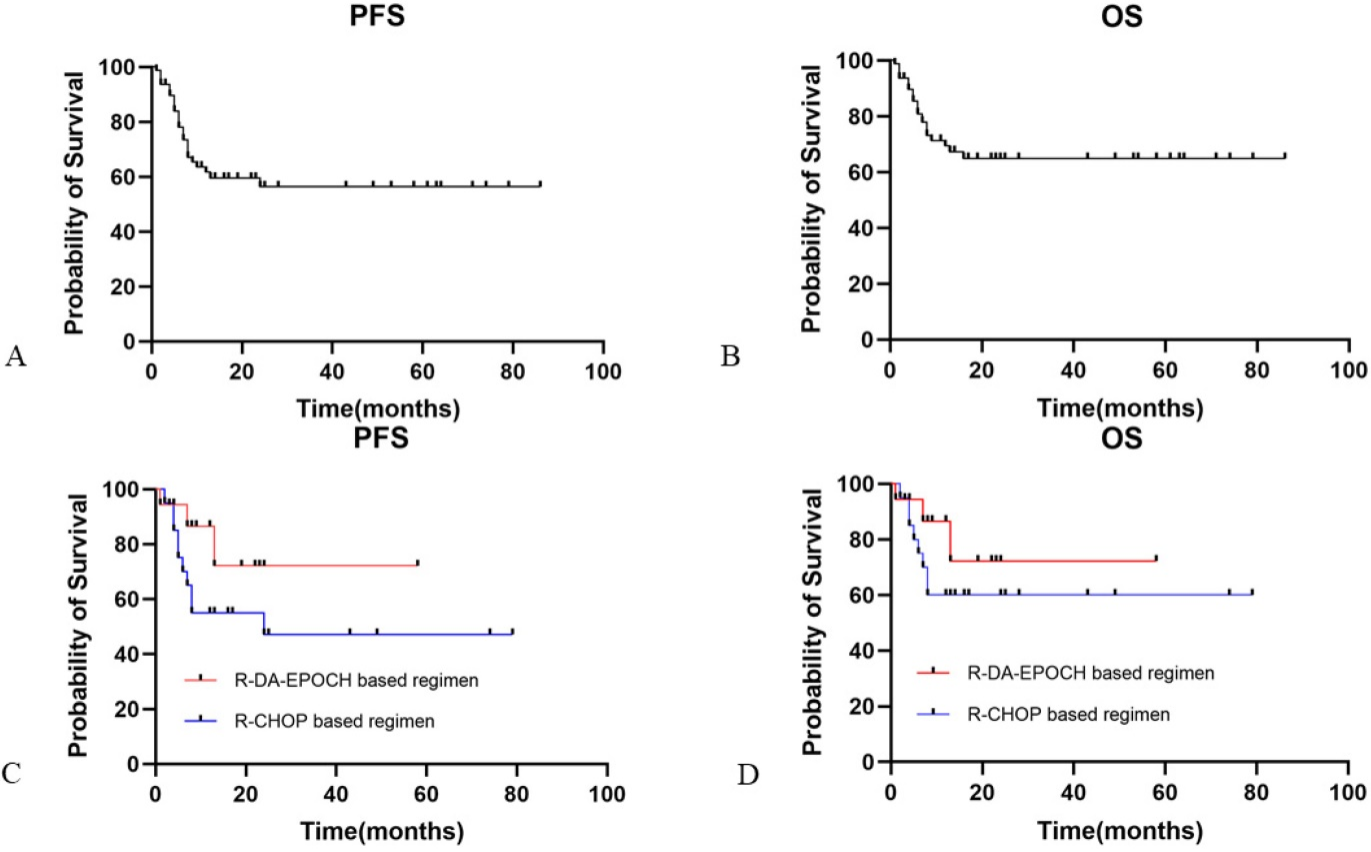
** Progression-free and overall survival in HIV-infected patients with DLBCL.** Panel A showed the PFS for HIV-infected DLBCL patients. Panel B showed the OS for HIV-infected DLBCL patients. Panel C showed PFS for HIV-infected DLBCL patients with R-DA-EPOCH based regimen and R-CHOP based regimen. Panel D showed OS for HIV-infected DLBCL patients with R-DA-EPOCH based regimen and R-CHOP based regimen. PFS: progression free survival; OS: overall survival; DLBCL: diffuse large B cell lymphoma.

The median PFS and OS for HIV-infected DLBCL patients in our study was not reached. The overall 2-year PFS and OS rates were 56.4% and 62.7%, respectively (Fig. 1A and 1B). ECOG performance status score ≥2 (HR: 6.01, 95% CI [2.71-13.33]) and IPI ≥3 (HR: 3.43, 95% CI [1.59-7.41]) were predictive of worse PFS, while ECOG performance status score ≥2 (HR: 5.5, 95% CI [0.60-50.19]) and IPI ≥3 (HR: 2.72, 95% CI [0.55-13.46]) were associated with worse OS. In contrast, in a multivariate Cox regression model, no HIV-related characteristics were associated with PFS or OS. Neither HBV nor HCV coinfection was associated with prognosis. Elevated plasma EBV DNA copy number was inclined to be associated with worse outcome (PFS and OS: p=0.08 and p=0.06, respectively).

When we evaluated the relationship stratified by aaIPI between treatment and clinical outcomes, for high aaIPI risk patients, the R-EPOCH-based regimen (n=18) was not significantly favored over the R-CHOP-based regimen (n=20) for either PFS (Fig. 1C; p=0.15) or OS (Fig. 1D; p=0.28).

## Discussion

Yunnan is located in Southwest China, bordering Myanmar, Laos and Vietnam, and has been regarded as the major site of the HIV infection epidemic for a long time 14, 15. Over the decades, people living with HIV/AIDS have been increasing in Yunnan, and it remains one of the provinces with the highest number of HIV-infected patients in China 18.

DLBCL is the most common pathological subtype of NHL in both the general population and in people living with HIV/AIDS 1-3, 19. A large database study shows that HIV infection continues to be an independent risk factor for death among patients with lymphoma 20. We analyzed the clinical features and survival outcomes of HIV-infected DLBCL in the setting of cART in our series. Consistent with other recent studies, HIV-infected patients with DLBCL presented with poor performance status, a high frequency of B symptoms, elevated LDH, advanced Ann Arbor stages, and high aaIPI scores 5, 6, 9, 17, 20. Moreover, bulky disease was frequent. EBV infection is found in approximately 10% of HIV-negative DLBCL 21; however, in our study, 66% of patients with HIV-positive DLBCL exhibited elevated plasma EBV DNA load, suggesting a higher rate of EBV coinfection. In contrast, the frequency of HBV infection in HIV-infected patients with DLBCL was similar to that of their HIV-uninfected counterparts reported in the Chinese population 11. Likewise, the frequency of HCV infection was close to that of HIV-negative DLBCL in the Caucasian population but lower than that in the Asian population 22, 23. Unlike a previous study in which HIV had been diagnosed a median of 15 years previously and in which nearly 80% of patients had received cART at DLBCL diagnosis 9, patients in our study experienced late HIV detection and late cART exposure. This may partially be attributed to decreased awareness of HIV testing and reduced initiative for medical care assistance in high-risk HIV populations.

Before the introduction of the anti-CD20 antibody rituximab, HIV-infected patients with DLBCL treated with CHOP therapy showed a similar response rate and survival compared to their HIV-uninfected counterparts 24, 25. Several retrospective studies of DLBCL patients with HIV infection treated with R-CHOP have been reported. In one study from Spain, HIV-infected DLBCL patients had a CR rate of 69% and a 5-year OS rate of 56%, similar to those of 81% and 74%, respectively, in HIV-uninfected patients 5. Similarly, in a report from France, survival outcomes of HIV-infected DLBCL patients (2-year PFS & OS: 81% and 81%) did not differ from those of HIV-uninfected patients (2-year PFS & OS: 71% and 83%) 9. Moreover, Sparana et al concluded that R-EPOCH was an effective regimen in HIV-associated NHL, achieving a CR rate of 73% 26. In our study, a total of 78 HIV-infected DLBCL patients treated between 2011 and 2019 were analyzed. Most patients received the CHOP±R regimen, and several patients were given EPOCH±R based on their disease status and financial situation. Additionally, a cooperative group prospective trial reported the safety and efficacy of autoSCT in HIV-positive lymphoma, including DLBCL 27. Here, five patients who underwent autoSCT after CR were lymphoma free. At the end of treatment, 58% of evaluable patients had achieved CR. The 2-year PFS was 56.4%, and the OS rate was 62.7%. The response rate and survival outcome appear to be lower than those in some studies. One of the reasons might be the decreased use of rituximab. Medical insurance has not covered the cost of rituximab until recent years. In addition, Yunnan is a remote province and relatively economically backward; thus, some patients could likely not afford their treatment, while a few exhibited low compliances and did not adhere to the recommended course of treatment. As a result, the high rate of loss to follow-up (20%) decreased the reliability of our results.

R-EPOCH is the preferred regimen for treating HIV-DLBCL, HIV-HHV8-positive DLBCL and HIV-primary effusion lymphoma (PEL) under current National Comprehensive Cancer Network guidelines based on multiple clinical trials and retrospective studies. In a pooled analysis of clinical trials for HIV-associated aggressive B-cell NHL comparing R-EPOCH (AMC034) to R-CHOP (AMC010), R-EPOCH resulted in superior outcomes compared to R-CHOP 28. In another study, treatment with R-EPOCH seemingly compared favorably to treatment with R-CHOP in HIV-associated DLBCL (p=0.05) 29. Recently, the histone deacetylase inhibitor vorinostat (VOR), combined with R-EPOCH, was reported to be tolerable and seemingly efficacious in patients with aggressive HIV-NHL 30. In our small sample size comparison, we did not observe a significant difference in survival between the R-EPOCH and R-CHOP regimens. However, all of these studies were conducted in a post hoc manner.

CNS involvement has been recognized to be more common in AIDS-related lymphomas 31. A retrospective review of databases from clinical trials showed that CNS involvement at baseline was not associated with shortened overall survival, but CNS relapse was associated with a reduced median OS of 1.6 months 31. Similarly, one patient in our study experienced CNS relapse and died soon thereafter. Although introduction of a prognostic model that is used for assessing the risk of CNS disease in DLBCL has guided CNS prophylaxis in the general DLBCL population, the value of this prognostic model in HIV-infected patients with DLBCL remains unclear. A survival difference was not observed with this prognostic model in our data, as most patients received intrathecal prophylaxis, suggesting the role of routine CNS prophylaxis in overcoming the unfavorable prognosis of high CNS-IPI.

It has been shown that HIV-related factors, such as low CD4^+^ cell count and prior history of AIDS, are no longer predictive of worse survival outcome in the cART era 32, 33, consistent with our results. We were not able to analyze the HIV viral load at diagnosis due to the lack of baseline level testing. It is one of the limitations of our study. On the other hand, levels of HIV-1 RNA load were negative in all subjects during chemotherapy. We found that unfavorable performance status and high IPI score were associated with poor outcomes. Interestingly, elevated plasma EBV DNA load exhibited borderline significance associated with poor outcomes. It has been widely accepted that EBV plays an important role in the pathogenesis of lymphomas, such as BL, cHL, DLBCL and natural killer (NK)/T-cell lymphoma 34. In the setting of HIV infection, EBV infection is more frequent and positive in 30-60% of cases compared to approximately 10% in general DLBCL patients 6, 13, 35, 36. We found that 66% of our DLBCL cases presented with elevated EBV DNA, consistent with the previously reported prevalence of EBV load in HIV-associated DLBCL 13. Its high frequency makes EBV a possible factor contributing to the development of HIV-associated lymphomas and is responsible for driving more aggressive behavior 35. In contrast to the fact that blood EBV DNA is not predictive of outcomes in HIV-associated HL 37, previous studies have demonstrated that tumor EBV infection status is an independent adverse predictive factor of survival among patients with HIV-infected DLBCL 38, 39. Muncunill et al. observed that plasma EBV load exerted a negative prognostic impact and could be used as an early predictor of HIV-related lymphoma 13. In this study, we showed that elevated whole blood EBV load was inclined to be an independent negative predictor for both PFS and OS, suggesting measurement of plasma EBV DNA for risk stratification. In HIV-negative DLBCL, HBsAg-positive patients exhibited worse clinical features and poor outcomes, and the presence of HCV conveyed inferior OS when accompanied by impaired liver function 11, 40, whereas our results indicated that HIV and HBV/HCV coinfection in DLBCL did not predict outcomes. Further studies with larger sample sizes are needed to more thoroughly investigate these findings.

A limitation of this study is the small sample size, although this is the largest series reported in China thus far. Moreover, missing data might have led to imprecise estimates. The chemotherapeutic regimens combined in some cases may also have led to difficulties in interpreting the results.

This study provides important real-world data on the clinical characteristics and outcomes of HIV-infected DLBCL patients in China. We conclude that survival outcomes remain poor, and additional therapeutic approaches are warranted in this population.

## Figures and Tables

**Table 1:** Clinical characteristics of HIV-infected patients with DLBCL

	N=78 (%)	Median (1^st^ -3^rd^ quartile)
Demographics		
**Gender**		
Male	67 (86%)	
Female	11 (14%)	
Age (years)		43 (38.0-52.0)
***HIV-related characteristics***		
**HIV transmission route**		
Intravenous drug use	6 (8%)	
Heterosexual	64 (82%)	
Homosexual	7 (9%)	
Mother to child	1 (1%)	
**Years of HIV infection at DLBCL diagnosis**		
<1 year	49(63%)	0.08 (0.03-0.24)
≥1 year	29(37%)	5.23 (2.40-9.05)
**CD4 cell count at DLBCL diagnosis (/μl)**		
<50	10 (13%)	25.0 (16.5-42.8)
50~199	37 (47%)	122.0 (92.0-157.0)
200~499	27 (35%)	278.0 (236.0-327.0)
≥500	4 (5%)	541.5 (524.0-553.0)
**cART initiation**		
cART prior to chemotherapy	26 (35%)	1.5 (0.4-4.4)
cART with chemotherapy (years)	52 (65%)	
***Lymphoma-related characteristics***		
**Cell-of-origin subtype**		
GC	49 (63%)	
Non-GC	29 (37%)	
**ECOG performance status score**		
0-1	20 (26%)	
2-4	58 (74%)	
LDH above normal	74 (95%)	360.5 (267.3-731.5)
B symptoms	58 (74%)	
**Extra-nodal sites**		
0	23 (30%)	
1	29 (37%)	
≥2	26 (33%)	
**Ann Arbor stage**		
I-II	15 (19%)	
III-IV	63 (81%)	
**aaIPI score**		
0-1	11 (14%)	
2-3	59 (76%)	
**IPI score**		
0-1	0	
2	1 (1%)	
3-5	7 (9%)	
**Bulky tumor (≥7.5 cm)**	50 (64%)	
Ki 67 >90%	22 (28%)	
BCL-2 expression positive by IHC^a^	30 (51%)	
Bone marrow involvement	15 (19%)	
**CNS-IPI score**		
0-3	51 (65%)	
4-6	27 (35%)	
***Other related characteristics***		
**EBV (whole blood)^ b^**		
<5×10^3^ copies/ml	20 (34%)	
≥5×10^3^ copies/ml	38 (66%)	4.04×10^4^ (1.06×10^4^-8.96×10^4^)
**HBV**		
HBsAg positive	8 (10%)	
**HBV-DNA load (copies/ml)**		
<5×10^2^	6 (75%)	
≥5×10^2^	2 (25%)	
**HCV**		
Anti-HCV IgG positive	8 (10%)	
**HCV-RNA load (IU/ml)**		
<5×10^2^	2 (25%)	
≥5×10^2^	6 (75%)	

HIV: human immunodeficiency virus; DLBCL: diffuse large B-cell lymphoma; cART: combination antiretroviral therapy; GC: germinal center; ECOG: Eastern Cooperative Oncology Group; LDH: lactate dehydrogenase; B symptoms: fever, night sweats, weight loss, fatigue, and swelling in lymph nodes; IHC: immunohistochemistry; CNS: central nervous system;^ a^: numbers of missing values,^ a^: 19. ^b^: numbers of missing values,^ b^: 20.

**Table 2:** Evaluation following chemotherapy in HIV-infected patients with DLBCL

	Interval evaluation^a^ (N=78)	At the end of treatment (N=62)
**Able to evaluate**	N=67	N=45
Complete response	14 (21%)	26 (58%)
Partial response	45 (67%)	6 (13%)
Stable disease	0	0
Progressive disease	3 (4%)	6 (13%)
Death^b^	5 (8%)	7 (16%)
**Unable to evaluate**	N= 11	N=17
Ongoing treatment	0	6
Having stopped treatment^c^	11	11

HIV: human immunodeficiency virus; DLBCL: diffuse large B-cell lymphoma; ^a^: Response evaluation after three or four chemotherapy cycles;^ b^: Death during the treatment; ^c^: Stop treatment before be able to evaluate response to chemotherapy could be attributed to personal willingness. Four cases end up in losing to follow-up and one patient committed suicide before or at the time of interval evaluation. Eight cases end up in losing to follow-up at the end of treatment.

**Table 3:** Cox univariable and multivariable analyses of PFS and OS in HIV-infected patients with DLBCL

	N	PFS				OS			
		Univariate analysis	P	Multivariate analysis	P	Univariate analysis	P	Multivariate analysis	P
		HR, 95%CI		HR, 95%CI		HR, 95%CI		HR, 95%CI	
**Gender**									
Male	67	0.75, [0.26-2.19]	0.56			0.61, [0.19-1.94]	0.31		
Female	11								
**Age (years)**									
≤60	70	1.12, [0.36-3.52]	0.85			0.87, [0.24-3.12]	0.82		
>60	8								
**Years of HIV infection at DLBCL diagnosis**									
<1 year	49	1.06, [0.50-2.25]	0.89			1.06, [0.46-2.43]	0.90		
≥1 year	29								
**CD4 cell count at DLBCL diagnosis**									
<200 (/μl)	47	1.55, [0.74-3.26]	0.25			1.60, [0.70-3.62]	0.27		
≥200 (/μl)	31								
**cART initiation**									
cART prior to chemo	26	0.59, [027-1.27]	0.21			0.50, [0.21-1.18]	0.16		
cART with chemo	52								
**Cell-of-origin subtype**									
GC	49								
Non-GC	29	1.71, [0.78-3.75]	0.14			1.78, [0.75-4.20]	0.16		
**ECOG performance status score**									
0-1	20								
2-4	58	6.01, [2.71-13.33]	**0.004**	4.47, [0.85-23.59]	0.08	10.54, [4.45-24.96]	**0.003**	5.50, [0.60-50.19]	0.13
LDH >UNL	74	1.16, [0.18-7.48]	0.88				0.28		
B symptoms	58	1.22 [0.54-2.76]	0.64			2.10, [0.87-5.05]	0.16		
**Extra-nodal sites**									
0-1	52								
≥2	26	0.88, [0.40-1.95]	0.75			0.90 [0.38-2.14]	0.82		
**Ann Arbor stage**									
I-II	15								
III-IV	63	2.50, [1.04-6.02]	0.11			3.15, [1.20-8.23]	0.10		
**IPI score (all patients)**									
0-2	25								
3-5	53	3.43, [1.59-7.41]	**0.010**	1.53, [0.45-5.21]	0.50	6.30, [2.74-14.47]	**0.004**	2.72, [0.55-13.46]	0.22
Bulky tumor (≥7.5 cm)	50	1.17, [0.53-2.57]	0.69			1.51, [0.64-3.56]	0.31		
Bone marrow involvement	15	1.47, [0.56-3.80]	0.37			1.08, [0.39-2.96]	0.87		
Ki 67 >90%	22	0.60, [0.27-1.33]	0.25			0.60, [0.25-1.42]	0.29		
**BCL-2 expression by IHC**	30								
positive	30	1.59, [0.66-3.82]	0.31			1.23, [0.49-3.27]	0.63		
negative	29								
**CNS-IPI score**									
0-3	51	0.88, [0.40-1.93]	0.74			0.79, [0.34-1.87]	0.58		
4-6	27								
**EBV (whole blood)^a^**									
<5×10^3^ copies/ml	20								
≥5×10^3^ copies/ml	38	2.09, [0.84-5.18]	0.08			2.75, [0.98-7.71]	0.06		
**HBV**									
HBsAg positive	8	1.06, [0.31-3.59]	0.93			1.20, [0.33-4.41]	0.76		
HBsAg negative	70								
**HCV**									
Anti-HCV positive	8	0.80, [0.22-2.96]	0.75			0.44, [0.11-1.79]	0.40		
Anti-HCV negative	70								

HIV: human immunodeficiency virus; DLBCL: diffuse large B-cell lymphoma; PFS: progression free survival; OS: overall survival; HR: hazard ratio; CI: confidence interval; cART: combination antiretroviral therapy; GC: germinal center; ECOG: Eastern Cooperative Oncology Group; LDH: lactate dehydrogenase; UNL: upper normal limit; B symptoms: fever, night sweats, weight loss, fatigue, and swelling in lymph nodes; CNS: central nervous system; ^a^: numbers of missing values,^ a^: 20.
